# Bio-Based Polymers for Environmentally Friendly Phase Change Materials

**DOI:** 10.3390/polym16030328

**Published:** 2024-01-25

**Authors:** Kinga Pielichowska, Katarzyna Nowicka-Dunal, Krzysztof Pielichowski

**Affiliations:** 1 Faculty of Materials Science and Ceramics, Department of Biomaterials and Composites, AGH University of Krakow, al. Mickiewicza 30, 30-059 Kraków, Poland; nowicka@agh.edu.pl; 2 Faculty of Chemical Engineering and Technology, Department of Chemistry and Technology of Polymers, Cracow University of Technology, ul. Warszawska 24, 31-155 Kraków, Poland; krzysztof.pielichowski@pk.edu.pl

**Keywords:** phase-change materials, bio-based polymers, thermal energy storage, shape stabilization

## Abstract

Phase change materials (PCMs) have received increasing attention in recent years as they enable the storage of thermal energy in the form of sensible and latent heat, and they are used in advanced technical solutions for the conservation of sustainable and waste energy. Importantly, most of the currently applied PCMs are produced from non-renewable sources and their carbon footprint is associated with some environmental impact. However, novel PCMs can also be designed and fabricated using green materials without or with a slight impact on the environment. In this work, the current state of knowledge on the bio-based polymers in PCM applications is described. Bio-based polymers can be applied as phase-change materials, as well as for PCMs encapsulation and shape stabilization, such as cellulose and its derivatives, chitosan, lignin, gelatin, and starch. Vast attention has been paid to evaluation of properties of the final PCMs and their application potential in various sectors. Novel strategies for improving their thermal energy storage characteristics, as well as to impart multifunctional features, have been presented. It is also discussed how bio-based polymers can extend in future the potential of new environmentally-safe PCMs in various industrial fields.

## 1. Introduction

A crucial problem of humanity within the 21st century is fossil fuels’ exhaustion and global warming that forces people to search for new solutions for energy saving and the production of energy from renewable sources. 

Phase-change materials (PCMs) are an important class of thermoresponsive materials used for the storage of thermal energy as sensible and latent heat. The application of PCMs in energy-related technical solutions can substantially impact the efficient use and conservation of sustainable and waste energy. Thermal energy storage in the form of latent heat provides a large density of energy storage with small temperature fluctuations during heat storage and release. As PCMs, different types of materials have been investigated including salts, salt hydrates, paraffins, and fatty acids, as well as polymeric materials usually characterized by solid–solid or solid–liquid phase transition [1,2,3]. They are usually modified with different materials to obtain shape-stabilized PCMs, encapsulated PCMs, or to improve some properties, e.g., thermal conductivity. 

Most of the currently applied PCMs are obtained from non-renewable sources and their environmental impact is connected with a certain carbon footprint. However, novel PCMs can also be designed and fabricated using green materials without or with a slight impact on the environment. 

Polymers are used in PCMs in different aspects, e.g., as PCMs themselves, for PCMs’ shape stabilization, or microencapsulation, but also as precursors in the carbonization process to obtain porous carbon materials that can be then applied for the shape stabilization of PCMs. Representative examples are poly(ethylene glycol) that is widely used as a polymeric PCM, and phenol formaldehyde and melamine formaldehyde resins, polyester, and acrylate resins that are widely applied for the microencapsulation of paraffin or fatty-acid-based PCMs [4]. On the other hand, for the shape stabilization of PCMs polyurethanes and polyethylene are utilized [5]. 

To decrease the negative impact of PCMs on the natural environment and carbon footprint, there is a search for “green’ solutions. One of the potential routes is the application of bio-based polymeric materials in the broad areas of PCMs and thermal energy storage by replacing (at least partially) synthetic polymers with bio-based ones [6,7,8]. Following this route it is possible to minimize PCMs environmental impact and carbon footprint in various sectors, such as building and energy sectors. 

Hence, this work reviews the current state of knowledge on bio-based polymers in PCM applications. Special attention has been paid to evaluation of properties of the final PCMs, and their application potential in various sectors. Novel strategies for improving their TES characteristics, as well as to impart multifunctional features, have been presented. The strategies for improving the thermal energy storage characteristics include attempts to (i) impart multifunctional features and (ii) to minimize PCMs environmental impact and carbon footprint. By using bio-based polymers it is possible to minimize negative environmental impact of PCMs. For this purpose bio-based polymers can be applied both as PCMs themselves and for PCMs shape stabilization. On the other hand, some additional but important features can be added by PCM modification with other additives to fabricate composite materials devoted for e.g., photothermal energy conversion, thermoelectric materials or materials for biomedical applications. It is also discussed how bio-based polymers can extend the potential of new environmentally safe PCMs in various industrial fields.

## 2. Bio-Based Polymers

According to the definition, bio-based polymers are polymers obtained from renewable resources, mostly from biomass [9]. At the end of the 20th century, the polymer industry was confronted with global warming, as well as the depletion of crude oil and petroleum-based products. Nowadays, bio-based polymers are a subject of intensive research, as they can play a crucial role in decreasing the carbon dioxide emissions causing global warming and using limited fossil resources [10]. One of the possible solutions of these problems was to use sustainable resources and materials instead of components derived from fossil resources [11,12]. The natural source of raw materials is biomass, and agricultural waste from, e.g., corn or potatoes could be a feedstock for numerous bio-based polymers [13]. 

However, in recent years, numerous research and industrial efforts have been made to develop new biotechnology and green chemical processes in order to preserve land devoted for food production. 

Bio-based polymers can be divided in three main groups:

- First class—polymers prepared from biomass, such as starch, cellulose, chitosan, chitin, sodium alginate, and natural rubber, including those polymers which are chemically modified;
- Second class—polymers obtained using microorganisms and plants, e.g., poly(hydroxyalkanoates) and poly(glutamic acid);
- Third class—synthetic polymers obtained from naturally derived monomers from renewable resources, e.g., bio-polyolefins, polylactide, poly(lactide-co-glycolide), poly(butylene succinate), bio-poly(ethylene glycol) PEG, bio-poly(ethylene terephthalate) [9,11,14].

**Figure 1:**
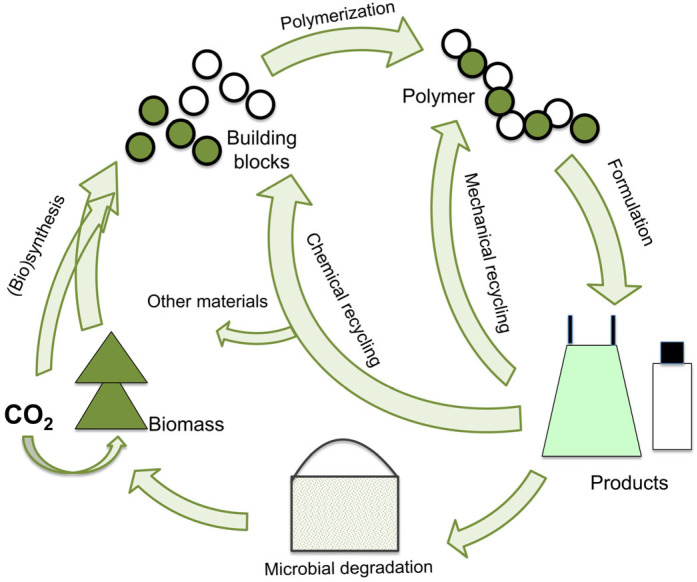
Presentation of the production of bio-based plastics and their recycling. Reprinted from [15] with permission from Elsevier.

Moreover, in agreement with the circular economy concept, the entire polymeric materials value chain, including materials’ design, recovery, and recycling after their lifetime, should be considered (Figure 1). 

Polymer materials based on renewable feedstocks are already being considered as carbon-neutral alternatives to fossil-based polymers, and renewable energy production plays a more and more important role in modern electricity systems.

**Figure 2:**
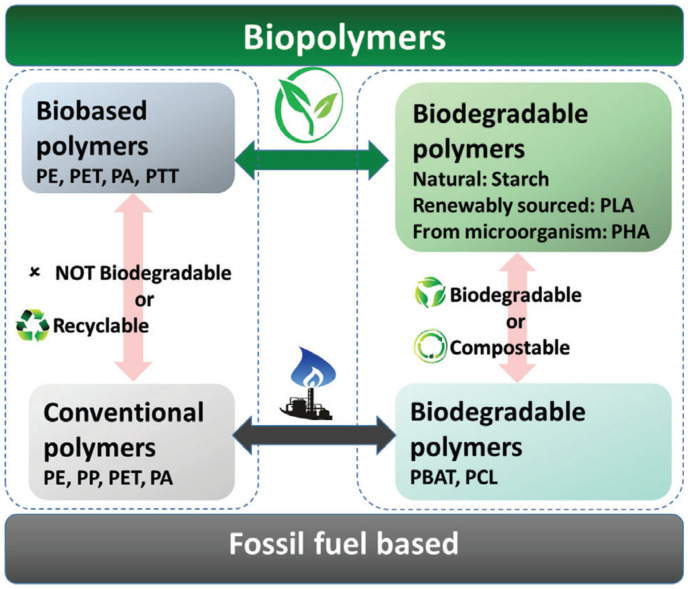
Classification of biopolymers. Reprinted from [16] with permission from Wiley.

Bio-based polymers can be biodegradable (polyhydroxyalkanoates, polylactide, starch, alginates) or non-biodegradable (bio-polyolefins, bio-based PEG). The classification of biopolymers is presented in Figure 2.

The biopolymers market has considerably grown recently up to the global production of about 2.4 million tons expected in the current year. However, it is still less than one percent of the total polymer production p.a. (390 million tons in 2021 [17]). Starch and starch-based polymers, polylactide (PLA), and polybutylene adipate terephthalate (PBAT) are the most commonly produced bio-based biodegradable polymers, while the group of bio-based non-biodegradable polymers includes PE, polyamide (PA), poly(ethylene terephthalate) (PET), and poly(trimethylene terephthalate) (PTT). Non-biodegradable biopolymers account for 44.5% of the annual production of biopolymers, while biodegradable biopolymers account for 55.5% of global production [16]. However, for PCMs applications, biodegradability is important only for some applications, e.g., in the biomedical field. For technical and industrial applications, usually non-biodegradable bio-based polymers are required. Some examples of the bio-based polymers currently available on the market are presented in Table 1; in Table 2, PCM systems with bio-based polymer components are shown. 

## 3. Bio-Based Polymers as Phase-Change Materials

Different types of polymers able to achieve a high degree of crystallinity can be applied as PCMs. In this section, polymers that are commercially available and produced using synthetic routes, which can also be obtained as bio-based polymers, have been described. In polymers, the key issue determining the temperature and heat of phase transition is the degree of crystallinity, which suggests that promising candidates for PCM will be polymers composed of linear macrochains, as well as those in which intermolecular forces enhance the arrangement of crystalline structures. In these polymers which could also be renewably sourced, crystallization occurs relatively easily and leads to the formation of regular crystalline forms. Hence, Kamimioto et al. [66] investigated the possibility of using high-density polyethylene (HDPE) as a PCM. HDPE melts at a temperature of 125–135 °C with a melting heat of up to 240 J/g. An additional advantage of using HDPE as a PCM is the possibility of crosslinking it; and then at the melting temperature of linear HDPE, a solid–solid transition of the crosslinked polymer is observed, associated with changes in macromolecular arrangement. Moreover, HDPE crosslinking (using physical or chemical methods) makes it possible to maintain shape stability at temperatures above the melting point. 

**Figure 3:**
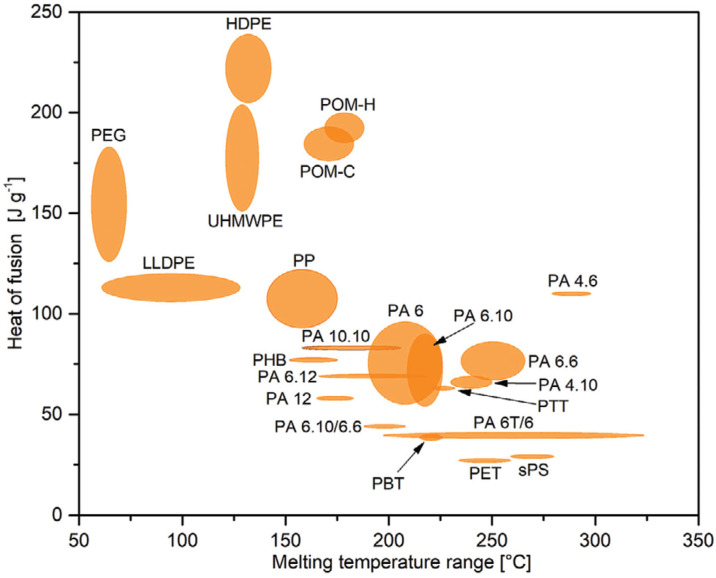
Overview of the melting behavior of examined semi-crystalline polymers generated via DSC when priorly cooled at −10 °C min^−1^ and heated at 10 °C min^−1^. The melting temperature range of each polymer type reflects the temperature range between the lowest T_Mon_ and the higher T_Mend_ of any polymer of the according polymer type. Reprinted from [68] with permission from Wiley.

In another work, Weingrill et al. [67] investigated high-density polyethylene (HDPE) long-term behavior (up to 7200 h) under air atmosphere. They found a minor loss in the storage capacity in thermo-oxidative conditions and concluded that HDPE is a good candidate material for polymeric PCM. Following this research program, the same research team analyzed the applicability of different semi-crystalline polymers, characterized by different phase-transition temperature ranges, as phase-change materials. As promising polymers for PCM applications, they proposed HDPE, PP, POM-H, and POM-C, as well as PA 4.6 and PA 6 [68]. The examined semi-crystalline polymers are presented in Figure 3. 

Another polymer considered as PCM was trans-1,4-polybutadiene (PB), which, depending on the temperature, occurs in two crystalline forms [69]. The solid–solid phase transition was detected at 78.1 °C, and the heat of this transformation was 144 J/g. Notably, PB melting occurs at a much higher temperature of 140 °C.

A promising group of macromolecular compounds to be applied as PCMs are those based on polyethers, especially polyethylene oxide (PEO). Aliphatic polyethers, also known as poly(alkylene oxides) or poly(alkylene glycols) (when the macromolecules are terminated with an –OH group) or polyalkyl oxides, are a group of polymers with a general formula –O–R–, where R consists of at least two methylene groups, formed by the ring-opening ionic polymerization of oxiranes and using initiating compounds containing an active hydrogen atom [70]. The most important representatives of aliphatic polyethers are PEO, poly(propylene oxide) (PPO), and polytetrahydrofurane (PTHF) [71,72].

Polyethers’ properties depend on the structure and average molecular weight. The first in the homologous series of aliphatic polyethers–poly(ethylene oxide) with an average molar mass not exceeding 600 is an odorless liquid with high viscosity at room temperature, while with a molar mass higher than 1000 it is a solid. The melting point ranges from 1–5 °C for PEO 400 to 65–67 °C for PEO 100,000—Table 3 [73]. 

Poly(trimethylene oxide) (PTMO) with three methylene units between ether oxygen atoms has a melting point ca. 35 °C, while polytetrahydrofurane with four methylene units between ether oxygen atoms shows a melting temperature ca. 60 °C. In aliphatic polyethers with an increasing number of methylene groups between ether oxygen atoms, the melting point rises to the value of ca. 130 °C, characteristic for linear polyethylene. All unsubstituted poly(alkylene oxides) are highly crystalline, with a degree of crystallinity, even up to 95% for PEO, but the presence of a side chain in the polyether chain causes in extreme cases a completely amorphous structure. PEO is soluble in water and polar organic solvents, and the chemical properties of polyethers are determined by the presence of ether bonds and hydroxyl end groups. Hydroxyl end groups undergo all reactions characteristic of alcohols, while ether bonds are highly resistant to hydrolysis [71,74]. The thermal properties of selected polyethers are shown in Table 4.

## 4. PCMs Encapsulation Using Bio-Based Polymers

In most applications, PCMs with a solid–liquid phase transition cannot be directly used due to the leakage above their melting point. Among the most common and effective methods to avoid the leakage, as well as to enhance other properties (such as mechanical ones), is encapsulation; in this process, PCM is sealed by selected tough materials that additionally allow for the enhancement of some PCM properties. The encapsulated PCMs usually consist of a polymer shell and core (the PCM itself). As a shell material, one can also use organic, inorganic, or hybrid organic–inorganic materials. The shell material selection should take into account its compatibility with PCM but can also aim at improving selected properties, e.g., dimensional stability or thermal conductivity [76,77]. The advantages and disadvantages of encapsulation protocols for PCMs are summarized in Table 5.

Among synthetic polymer-based shell materials, melamine-formaldehyde (MF) resin, poly(urea-urethane) (PUU), polyurea (PU), urea-formaldehyde (UF) resin, acrylic, resins and polystyrene are vastly used. However, numerous bio-based polymers have been used as shell materials for PCMs, including gum Arabic, chitosan/gelatin, and agar/gelatin/gum Arabic compositions [77]. 

Hence, Deveci and Basal [78] prepared microcapsules with an n-eicosane core as a PCM and silk fibroin (SF) and chitosan (CS) as a shell material by the complex coacervation method. Sphere-shaped microcapsules were successfully obtained, and the EDS analysis confirmed the effective incorporation of n-eicosane in the microcapsules. The microcapsules obtained at an SF/CS ratio of 5 possessed a dense and nonporous wall, while for the ratio of 20 an inner layer with a smoother surface and a rough outer surface with a spongelike structure were observed. It has also been found that the microencapsulation efficiency affected the n-eicosane content in such a way that a high n-eicosane content improved the microencapsulation efficiency, especially at higher SF/CS ratios. 

Phadungphatthanakoon et al. [48] encapsulated n-eicosane (C20) into a blend of methyl cellulose (MC) and ethyl cellulose (EC). They observed up to a 24 and 29% increase in the heat of phase transition during crystallization and melting for the microparticles (C20/EC/MC with a 9% EC/MC blend content), respectively. Next, C20-loaded EC/MC microparticles were incorporated into natural rubber latex to obtain natural rubber-based composites with an acceptable thermoregulation property and improved mechanical properties.

In another development, PCM microcapsules based on n-nonadecane as the core material and sodium alginate (SA) as the shell material were fabricated by Moghaddam et al. [6] using a melt coaxial electrospray. It has been observed that the microcapsule size was increased with the SA concentration, while smaller microcapsule diameters and a more homogenous dimensional distribution were found for SA concentrations of 1.5% (*w*/*v*). A decrease in the needle-to-collector distance from 20 to 5 cm caused mean microcapsule size decrease from 480 to 275 µm. A further reduction in the working distance up to 3 cm allowed them to obtain spherically shaped microcapsules with diameters lower than 100 µm. The obtained results confirmed that for microcapsules with diameters smaller than 100 µm, the n-nonadecane encapsulation ratio was 56 ± 5%. 

Fashandi and co-workers [54] investigated microencapsulation process of palmitic acid (PA) of vegetable origin in bio-based PLA shells using solvent evaporation and oil-in-water emulsification methods. They revealed that higher PA content lead to higher core content, and an increase in microPCM’s size. This was connected to the higher viscosity of the oil phase. The obtained results exhibited that PVA is a good emulsifier, and that proper PVA concentration choice make it possible to control the average size of microPCM capsules.

**Figure 4:**
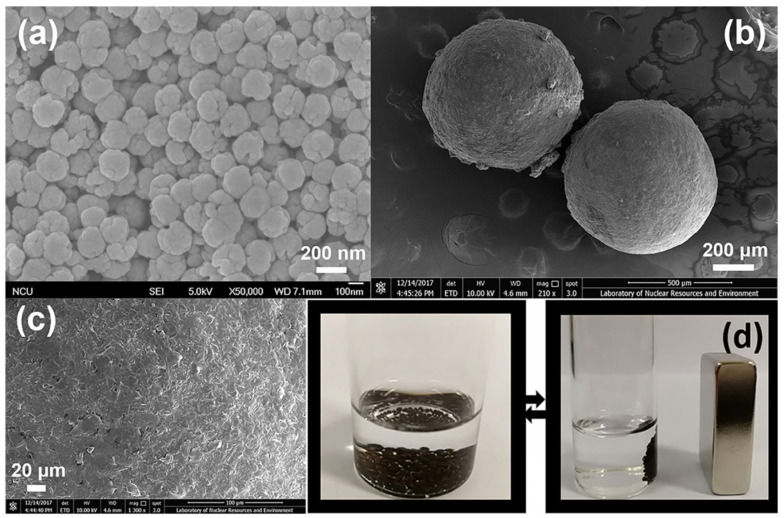
(**a**) SEM micrographs of Fe_3_O_4_ nanoparticles, (**b**) SEM micrographs of M-PCM-20-CTS, (**c**) high-magnification SEM micrographs of M-PCM-20-CTS, (**d**) the digital photos of an aqueous suspension containing M-PCM-20-CTS before and after magnetic separation. Reprinted from [24] with permission from Elsevier.

Multifunctional magnetic microcapsules based on n-eicosan and chitosan were fabricated by Chen et al. [24]. They used multi-emulsification and crosslinking approaches, as showed in Figure 4. The obtained microcapsules exhibit a regular spherical morphology with a uniform diameter of 500 μm and a heat of phase transition of up to 80 J/g. 

Tangsiriratana et al. [56] studied microencapsulated PCMs composed of gelatin/gum Arabic (shell) and a sugarcane wax–Al_2_O_3_ composite (core), obtained via complex coacervation. The authors found that the heat storage-dissipation performance and thermal stability of PV panels with the sugarcane wax-based PCM composite in solar panels was influenced by its thickness. Additionally, the PV panels’ front-facing glass peak temperature was reduced by ca. 4% when the layer thickness of the encapsulated PCM was increased from 4 to 7 mm. 

**Figure 5:**
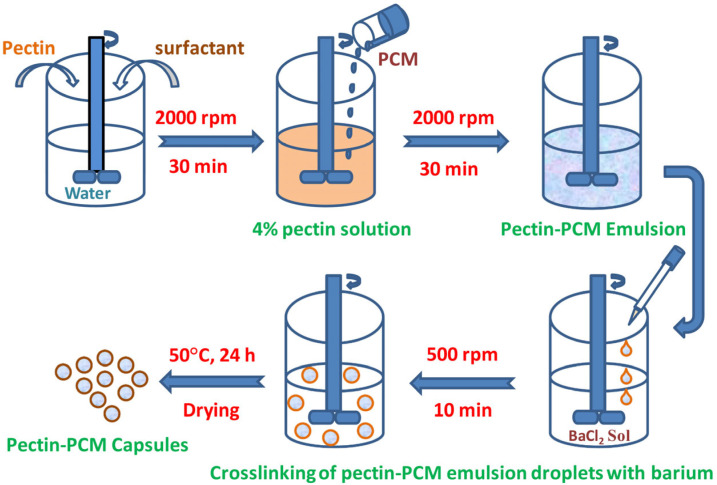
Schematic diagram of a pectin–PCM capsules preparation method through ionic gelation. Reprinted from [46] with permission from Elsevier.

Reddy et al. [46] obtained PCMs based on caprylic acid, hexadecane, and butyl stearate encapsulated in the barium-crosslinked pectin shell following the ionic gelation protocol as a core—Figure 5. 

Pectin/PCM capsules with diameters lower than 2 mm were obtained with an encapsulation efficiency of 83.66 wt.%, 83.21 wt.%, and 84.39 wt.% for hexadecane, butyl stearate, and caprylic acid as the PCM, respectively. They were characterized by melting enthalpies of 184.89 kJ/kg, 116 kJ/kg, and 118 kJ/kg. It was also revealed that pectin encapsulation caused an improvement in the thermal stability of encapsulated PCMs by 70–130 °C, compared to pristine PCMs. 

## 5. PCMs Shape Stabilization Using Bio-Based Polymers

Another method to avoid the leakage of PCMs above their melting temperature is shape stabilization, usually by using porous materials. The shape-stabilization method with porous lightweight materials can limit PCM leakage during the phase transition, but can also improve selected properties such as the thermal conductivity [79].

PEO blends with selected polymers of natural origin or their derivatives—cellulose, cellulose acetate, cellulose ether, carboxymethoxycellulose, and potato starch—were obtained and characterized by the DSC and IR techniques. It has been shown that the phase transformations of the solid–solid type occur in PEO/natural polymer systems and, in the case of selected blends (PEO/cellulose acetate 1:1 *w*/*w* and PEO/CMC 1:1 *w*/*w*), a synergistic effect appears; however, the obtained systems are characterized by a relatively low phase-transition heat. The shape-stabilization effect was attributed to intermolecular hydrogen interactions between ether oxygen in PEO chains and cellulose (or its derivative) hydroxyl groups, or PEO hydroxyl end groups and oxygen atoms in cellulose macrochains [7,40].

Zhao et al. [50] prepared n-octadecane/silk composite fibers designed as form-stable PCMs by the emulsion–electrospinning method. Microscopic observations showed that the composite fibers are characterized by a cylindrical shape, uniform diameter, and smooth surface, while the DSC results revealed a high thermal energy storage capacity with reversible phase-transition behavior and a good thermal reliability after thermal cycling.

A eutectic mixture of TD and MA was incorporated into hydroxylpropyl methyl cellulose (HPMC) by Qu et al. [45]. The obtained results confirmed that a TD–MA eutectic mixture was distributed uniformly in HPMC, and a form-stable PCMs were obtained. Leakage tests revealed that the absorption efficiency of TD-MA by HPMC was nearly 50%. The thermal properties investigations showed that the temperatures of melting and freezing were 34.61 and 31.09 °C, and the heat of phase transition was 102.11 and 84.58 J/g, respectively. Importantly, the TD-MA/HPMC exhibited a good thermal stability and reliability performance.

**Figure 6:**
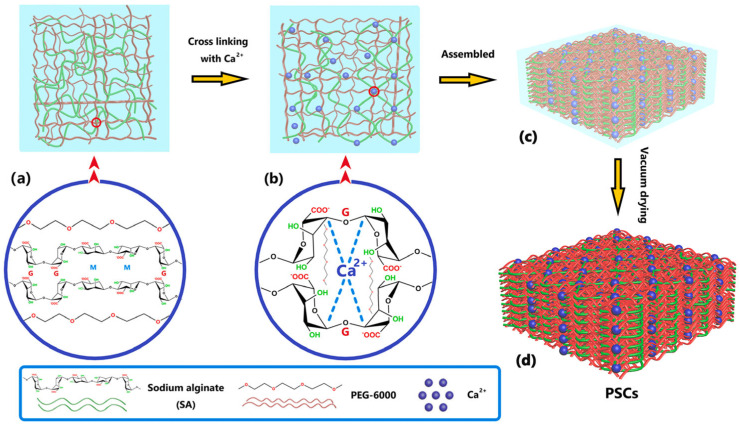
Schematic illustration of the PSCs synthesis process. Reprinted from [34] with permission from Elsevier.

Liu et al. [34] reported on shape-stabilized PCMs based on PEG 6000 and SA, prepared via a sol–gel method. Ca^2+^-crosslinked sodium alginate was modified with PEG—Figure 6. The PEG loading rate in the PEG/SA composites (PSCs) was 93% with a phase-transition enthalpy of up to 156.8 J/g and a satisfactory shape stability.

PEG-based PCMs shape-stabilized by chitin nanofibers (CNFs) were obtained by Wijesena et al. [23]. Fibrous chitin from crab shells was characterized by a diameter of several tens of nanometers and lengths of up to few micrometers. The best system remained opaque (transmittance ∼3.5%) below the melting temperature of PEG, while above the melting temperature it was transparent with a transmittance value of ∼88%. 

Pinto and co-workers [64] prepared shape-stabilized PCMs based on bacterial cellulose nanofibers (BC) with reduced and non-reduced graphene oxide (GO) and microencapsulated paraffin as PCMs in the foam-like form. They revealed that the presence of GO enhanced the fire-retardancy and dimensional stability of the obtained system. 

Capric acid and lauric acid encapsulated in SA were integrated with oakum fiber in a plaster matrix by M’ghari et al. [55]. The obtained results confirmed a good compatibility between the components and a sufficient chemical and thermal stability; a phase-change temperature of 16.54 °C and heat of phase transition of 35.18 J/g were determined after thermal cycling. The designed composite material can be considered, due to the high heat capacity, low cost, and proper phase-transition temperature, for energy storage in the building sector.

**Figure 7:**
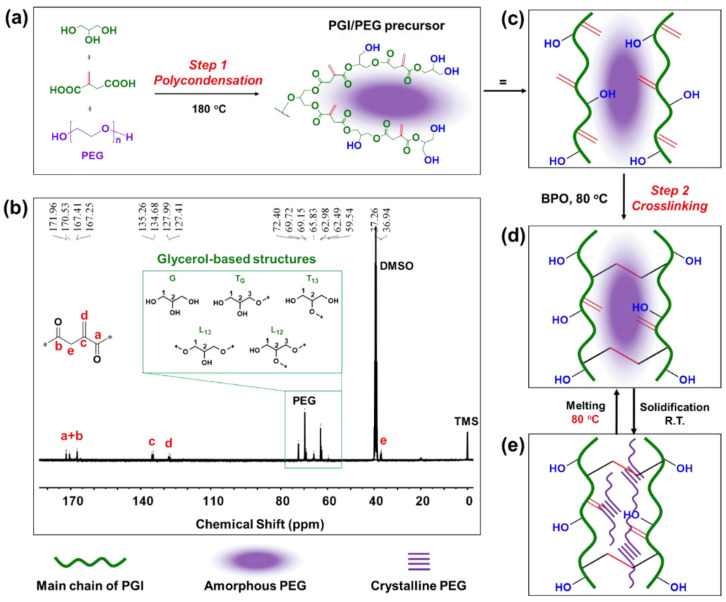
(**a**) Synthesis of a PGI/PEG precursor by polycondensation (Step 1), (**b**) 13C NMR full spectrum of PGI/PEG (PGI-45) (before curing), (**c**) Schematic diagram of PGI/PEG precursor with free vinyl and -OH groups, (**d**) Cured PGI/PEG induced by the BPO initiator at 80 °C (Step 2) and (**e**) structure illustration of the reversible phase change. Reprinted from [35] with permission from Elsevier.

Bio-based poly(glycerol-itaconic acid) (PGI) as a support for PEG-based form-stable PCMs was proposed and tested by Yin et al. [35]. The PGI/PEG precursor has been synthesized by solvent-free polycondensation (Figure 7), where PEG as a PCM was encapsulated within the crosslinked PGI. Interestingly, PGI can entrap up to 72.67% of PEG 6000 with an excellent form stability and phase-transition enthalpies of up to 61.8–86.9 J/g. The form stability of the PGI/PEG system was supported by intermolecular hydrogen bonds, and the phase-change temperature and heat of PCMs can be controlled by both the PEG load and its average molar mass.

Ahn et al. [62] fabricated polymer fibers with a microencapsulated PCMs load up to 80 wt.% by incorporating commercially available PCM microcapsules into cellulose acetate, polyethersulfone, or cellulose and processing by solution-spinning. The obtained fibers were characterized by ca. 95% of thermal storage capacity, compared to the microcapsules used. Importantly, the DMA results show some relationships between the mechanical stability of PCM-polymer fibers and PCM load.

PEG2000–10,000 was impregnated into CNT/cotton yarn (CCY) and coated by electrospun PAN [36]. The obtained yarn contained conductive yarn with PEG located between the cotton fibers, and it was characterized by a heat of phase transition in the range of 126–150 J/g. 

Singh et al. [42] proposed to use PCM beads to provide thermal buffering to a chocolate-containing box subjected to higher temperatures. For this purpose, 1-dodecanol was incorporated into barium alginate beads by ionotropic gelation and coated with a polyurea shell. The latent heat of the microcapsules without the polyurea coating was ca. 149 J/g, while after polyurea coating had been applied it amounted to ca. 76 J/g. The obtained beads were introduced to the inner walls of the packaging box to keep the temperature around 25 °C; the performed tests showed that the developed capsules could maintain the chocolate temperature at 25 °C by 86 min.

Meng et al. [59] used sodium alginate for the stabilization of sodium sulfate decahydrate (Na_2_SO_4_·10H_2_O) (SSD); the SA/SSD systems were prepared by blending SSD with SA at different concentrations. The best composite PCMs exhibited a phase-change enthalpy of up to 160 J/g, and the stabilization effect was attributed to the ionic and hydrogen bond interactions between SA and SSD.

Electrospun phase-change nanofiber films based on lignin were obtained by electrospinning by Niu et al. [26] using sodium lignosulfonate (SLS), poly(vinyl alcohol) (PVA), and PEG. In the proposed system, the SLS/PVA mixture acted as a supporting matrix; additionally, SLS is a photothermal material and interacts with PEG via hydrogen bonds and electrostatic forces. The obtained nanofibers exhibited a good solar-to-thermal energy conversion and storage ability with a latent enthalpy of fusion of 42.16 J/g, whereby the solar-to-thermal energy conversion and storage efficiency amounted to ca. 18%. 

Wang et al. [52] studied a composite PCM based on silk fiber (SF) for wearable personal thermal management [52]. For preparing the PCM composite, natural silkworm cocoons and capric acid (CA) were used. The performed investigations revealed that SF and CA were physically bonded, and the crystal structure of CA was not changed by SF. The heat and temperature of the phase transition of SF/CA were 123.4 J/g and 30.6 °C, respectively, and the SF/CA composite showed a good shape and thermal stability. 

Lignin–g–poly(ε-caprolactone) as a form-stable PCM was investigated by Lee et al. [60]. The copolymer was synthesized by grafting of PCL with alkaline lignin via ring-opening polymerization, while the physical mixtures of lignin with PCL were also prepared for comparative study. DSC data revealed that the melting temperature of lignin–g–PCL was ca. 51.3 °C and the latent enthalpy of phase transition was ca. 61 J/g. Tests also revealed its good thermal reliability and stability after a thermal cycling test of up to 100 cycles. 

Liu and co-workers [41] prepared and tested coaxial fiber membranes (CFMs) with PEG stabilized by bio-based PLLA, prepared using coaxial electrospinning. The performed studies indicated that CFMs with a PEG content of 49 wt.% show a good energy storage ability and thermal stability. Moreover, in comparison with the PLLA membrane without PEG, the thermal insulation efficiency was improved more than 21%. 

In another work, Li et al. [25] obtained porous capsules as shape-stable PCMs based on PEG, and a cellulose polyelectrolyte complex synthesized using carboxymethyl cellulose (anionic polyelectrolyte) and cellulose 6-(N-pyridinium)hexanoyl ester (cationic polyelectrolyte). To improve the thermal conductivity and photothermal conversion efficiency, MWCNTs or GO were introduced. Capsules with a core-shell structure exhibited a thermal energy storage ability with the latent enthalpy of phase transition 142.2 J/g and promising form-stable properties, thermal stability, and photothermal conversion performance. Erythritol (ET)-grafted polylactic acid (PLA) (ETPLA) polymers were in situ synthesized as shape-stabilized PCMs by Lee et al. [61]. The thermal conductivity of ETPLA was improved by modification with an oxidized carbon fiber (CF-OH) and aluminum nitride (AlN). The obtained PCM composite exhibited a thermal conductivity of 4.25 W/mK, much better mechanical properties compared to pure ET, and a very high latent enthalpy of fusion of up to 170.7 J/g, making the fabricated PCM composites a good candidate for thermal management systems of electronic devices.

Baniasadi et al. [27] used PCL for the shape stabilization of PEG nanofiber mats obtained by electrospinning. Additionally, gelatin and curcumin were applied to enhance the biomedical performance of the fabricated textiles. Microstructure analysis of the mats indicated nanofibers with randomly oriented morphology and diameters in the range of 220–270 nm. Mats were also characterized by good water absorption and mechanical properties. The heat of phase transition was 61.7 J/g with a good thermal reliability over 100 heating–cooling cycles. Moreover, the textiles modified with curcumin show a significant antioxidant activity, indicating a great potential application as a biomedical dressing that reduces inflammation and exerts a cooling effect.

**Figure 8:**
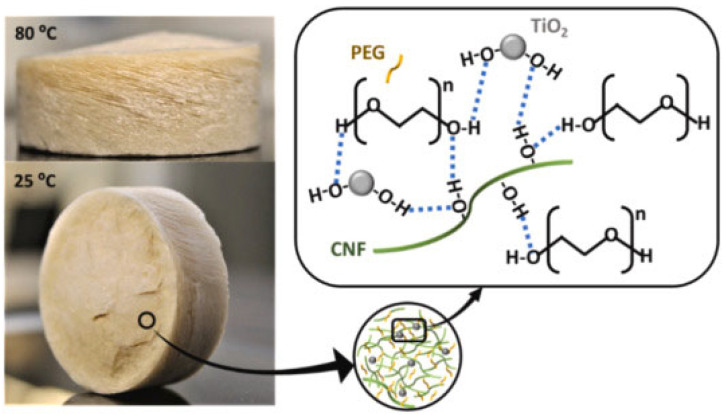
Form stability and leakage-proof phase transition of a phase-change nanohybrid (PCN-L3) composed of PEG (74 wt.%), lignin-CNF (24 wt.%), and TiO_2_ (2 wt.%) through intermolecular interactions and hydrogen bonding. Reprinted from [32] with permission from Elsevier.

Highly porous PEG-based materials with modified cellulose nanofibrils (CNF), lignin-containing CNF (LCNF), or acetylated CNF (ACNF), containing titanium dioxide, were prepared by Le et al. [32]—Figure 8. The PEG/CNF hybrid was characterized by a very low density in the range of 0.022–0.043 g/cm^3^ and a high latent enthalpy of phase transition of up to 204 J/g. The obtained system was tested as a material for buildings’ thermal management, and it was found that under excess solar heating, the PCM composite provided a good insulative protection and exceptional acoustic absorbance; modification with titanium dioxide nanoparticles improved the thermoregulating abilities.

In PEG20,000/PLA/graphitic carbon nitride (g-C3N4) composite PCM, prepared using the confinement method, PEG hinders the crystallization of PLA, and it was confirmed using the XRD method. The authors found that the incorporation of PEG changed the number of diffraction peaks of PLA, suggesting disrupted PLA crystallization, and in consequence a decrease in hardness [29]. The composite PCM exhibited a heat of phase transition of 106.1 kJ/kg for PLA:PEG:g-C3N4 with a mass ratio 3:6:1, and an increased thermal conductivity compared to PLA. 

Kizildag [65] used microcapsules with melamine–formaldehyde shells and paraffin cores as PCMs incorporated into pullulan solution. Pullulan films were obtained by the film-casting method of prepared dispersion (75 wt.% of PCM), and they showed an enthalpy of phase transition of 104.85 J/g during heating and 103.58 J/g during cooling, as well as a good thermal reliability after thermal cycling. 

## 6. Conclusions

Bio-based polymers are currently considered as future candidates for the design and manufacturing of modern phase-change materials. The application of bio-based polymeric materials allows us to minimize PCMs’ environmental impact and carbon footprint, especially in the energy- and materials-consuming building and energy sectors. Even a partial replacement of synthetic polymers with bio-based ones makes it possible to decrease the negative impact of PCMs on the natural environment and carbon footprint. Current research works focus on two topics: (i) PCMs encapsulation using bio-based polymers, and (ii) PCMs shape stabilization by bio-based polymers. In the former area, to avoid leakage, as well as to protect and enhance PCM properties, various encapsulation strategies have been developed, and the most up-to-date trend is the use of bio-based components in the (micro)capsules preparation process. It has been found that the application of bio-based polymers, for instance cellulose and its derivatives, enables the fabrication of encapsulated PCMs with good thermoregulation abilities and acceptable mechanical properties. Interestingly, microencapsulated PCMs composed of gelatin/gum Arabic (polymer shell), and sugarcane wax–Al_2_O_3_ composite (core material) have already been tested in solar panels, and a desired reduction in the front-facing glass temperature was observed. In the second area, i.e., PCMs shape stabilization, bio-based polymers are applied to limit PCM leakage during the phase transition, but can also improve selected properties, such as thermal conductivity, as well as shape stability which is of primary importance. In synthetic/natural polymer blends, e.g., PEO/cellulose (derivatives), intermolecular hydrogen interactions between oxygen atoms in PEO chains and hydroxyl groups in cellulose chains (or its derivatives), or hydroxyl end groups in PEO chains and oxygen atoms in cellulose chains, were found to improve the shape stabilization effect. On the other hand, the physical modification of PCMs has also been performed to impart new features to the shape-stabilized systems; e.g., the incorporation of graphene oxide remarkable improved the fire-retardancy of bacterial cellulose-based PCM. In another development, PCMs modified by multi-walled carbon nanotubes were found to provide an advantageous light-to-thermal conversion performance. 

To conclude, bio-based polymers are a vast group of materials that can be applied in the design and manufacturing of environmentally friendly phase-change materials with a reduced carbon footprint. Biopolymers can be chemically or physically modified to give them new features, and such new (composite) materials will gradually replace components produced from non-renewable sources and thus contribute to the development of a future advanced ‘green’ economy.

## Figures and Tables

**Table 1:** Bio-based polymers.

Biopolymer	Biobased Content (%)	Major Producers	Applications	Biodegradability	Ref.
Starch	100	Novamont; Futerro; Biome	Flexible packaging, consumer goods, agriculture	Yes	[18,19]
PLA	Up to 100	NatureWorks; Total Corbion; Shimadzu Cor.; Toyobo; Evonik,	Flexible packaging, rigid packaging, consumer goods	Yes	[18,19]
PHA	100	Bio- On; Kaneka; Tepha; Danimer Scientific; Newlight Technologies; Yield10 Bioscience, Tianjin GreenBio Materials,	Flexible packaging, rigid packaging	Yes	[18,19]
PHB	n/a	Mitsubishi Chemical; Showa Denko K.K.; SK Chemicals; PTT MCC Biochem	Flexible packaging, rigid packaging	Yes	[19]
PBS	Till 100	Showa Denko K.K.; SK Chemicals; Mitsubishi Chemical	Flexible packaging, agriculture	Yes	[18,19]
PBS	Til 50	Natureplast;	Flexible packaging, rigid packaging, regular consumption goods, horticulture/agriculture	Yes	[20]
PEG	Til 100	Sirius; Croda; Solvay; Clariant IGL Specialty Chemicals; BASF; Helvetic; Bioscience	Consumer goods	Yes	[18]
PBSA	Till 100	Showa Denko K.K.; SK Chemicals;	Flexible packaging, agriculture	Yes	[18,19]
PBAT	Till 50	BASF; Zhuhai Wango Chemical Co.; JinHui ZhaoLong; Eastman; Novamont	Flexible packaging, rigid packaging, agriculture	Yes	[18,19]
PCL	n/a	Perstorp until 2018, acquired by Ingevity	Consumer goods	Yes	[18,19]
PTT	27–37	Dupont	Fibers (including woven and non-woven), consumer goods	No	[18,19]
PEF	Till 100	Avantium; Corbion; BASF; DuPont, Synvina	Packaging: replacement for PET	No	[18,19]
PE	n/a	Braskem; Neste; LyondellBasell	Flexible packaging, rigid packaging, building and construction	No	[19,21]
PP	30	Borealis; Neste; LyondellBasell	Flexible packaging, rigid packaging,	No	[21]
PA	Till 100	Natureplast; Technoform; Nexis Fibers; Acro; Avient; Fulgar; Akeme; Hengshui	Technical parts, regular consumption goods, sports and leisure, transport	No	[20,22]
PET	Till 75	Toray Industries, The Coca-Cola Company, M&G Chemicals; Virent; PepsiCo; Toyota Tsusho	Rigid packaging	No	[18,19]

**Table 2:** PCM systems with bio-based polymer components.

PCM	Bio-Based Polymer	T_m_ [°C]	T_c_ [°C]	Heat of Melting (J/g)	Heat of Freezing (J/g)	Ref.
PEG 1000	Chitin nanofibers (CNFs) from crab shells of *Portunus pelagicus*	N/A	24.47	N/A	109.65	[23]
PEG 1000	Chitosan with a deacetylation degree of 80–95%	40	73	24/31	75	[24]
PEG 1000	Microcrystalline cellulose (MCC)	65.47	142.2	42.32	137.4	[25]
PEG 1000	Lignin	45.20	42.16	43.06	37.88	[26]
PEG 1000	Gelatin from bovine skin (Type B)	33.2	26.4	61.7	52.6	[27]
PEG 2000	Spongy-like porous carbon derived from eggplants	61.55	149.00	19.90	138.14	[28]
PEG 2000	PLA	64.54 174.15	37.51 68.73	189.80 16.3	96.43 11.32	[29]
PEG 4000	Dried pomelo peel foam	62.7	158.1	41.5	156.1	[30]
PEG 4000	Enzymatic lignin	61	41	168.7	165.12	[31]
PEG 4000	Cellulose nanofibrils (CNF) never dried and partially delignified birch pulp fibers ACNF: The CNF was modified by heterogeneous acetylation LCNF: lignin-containing nanofibril suspension	58.4 58.2	N/A	115.1 142.6	109.7 137.2	[32]
PEG 6000	Gum tragacanth	55.3	106.8	39.2	111.3	[33]
PEG 6000	Ca^2+^- Crosslinked SA	N/A	156.8	N/A	150.3	[34]
PEG 6000	PGI	58.35	86.93	14.15	83.65	[35]
PEG 2000 to PEG 10,000	Cotton composite yarn (PPCCY) was fabricated by impregnating PEG2000–10,000 into CNT/cotton yarn (CCY) and coating electrospun PAN on its surface Composite PPCCY with various PEG types: PEG 2000 PEG 4000 PEG 6000 PEG 10,000	PEG 2000–56.5 PEG 4000–65.3 PEG 6000–62.9 PEG 10,000–66.3	PEG 2000–135.3 PEG 4000–136.0 PEG 6000–150.8 PEG 10,000–126.0	PEG 2000–29.8 PEG 4000–35.8 PEG 6000–35.6 PEG 10,000–39.5	PEG 2000–128.8 PEG 4000–128.4 PEG 6000–150.9 PEG 10,000–125.3	[36]
PEG 4000 to PEG 10,000	To produce a PDA layer on the surface of the freeze-dried radish, the self-polymerization of dopamine was initiated	64.77	161.52	44.32	158.41	[37]
PEG 10,000	CAC	62.3	60.6	37.8	58.7	[38]
Isocyanate terminated PEG 6000	Pomelo peel flour (PPF) with an average particle size of about 55 μm	62.5	182.3	36.1	173.5	[39]
PEO	Cellulose (microcrystalline powder 20 mm, degree of polymerization: 229) CAC (40% substitution acetyl groups) CMC (60% substitution, powder *<* 400 mm) CET (Cellulose ether; 30% substitution, powder 350 mm) PEO/CEL PEO/CMC PEO/CAC PEO/CET	PEO/CEL–63.4 PEO/CMC–58.4 PEO/CAC–62.7 PEO/CET–64.7	PEO/CEL–134.7 PEO/CMC–140.2 PEO/CAC–156.3 PEO/CET–156.8	PEO/CEL–32.5 PEO/CMC–36.6 PEO/CAC–27.6 PEO/CET–37.2	PEO/CEL–127.3 PEO/CMC–138.0 PEO/CAC–152.8 PEO/CET–153.3	[40]
PEO	Potato starch	67.3	96.9	33.5	94.6	[7]
PEO with a viscosity average molecular weight (Mv) of 1 × 10^5^ g/mol	PLLA	60.95	58.79	46.31	57.59	[41]
1-Dodecanol	SA	25.69	149.15 ± 3	14.65	147.79 ± 3	[42]
Dodecane	CS (low M_W_ with a degree of deacetylation of 75–85%), to obtain the biochar papermill sludge, was used as biomass	−9.9	N/A	91	N/A	[43]
1-Dodecanol from coconut extract (70 wt.%)	Bio-based transparent wood: delignified and succinylated birch wood	25.2 ± 1	141 ± 5	19.3 ± 0.6	128 ± 7	[44]
TD and MA	Hydroxylpropyl methyl cellulose (HPMC).	34.61	206.45	31.09	204.59	[45]
Hexadecane, butyl stearate, caprylic acid	Poly(D-galacturonic acid methyl ester)	H5—23.99 B5—21.60 C5—18.50	H5—184.89 B5—116.03 C5—118.04	H5—1.49 B5—10.34 C5—−19.85	H5—185.89 B5—118.26 C5—114.42	[46]
n-Nonadecane	Alginic acid sodium salt	32.10	28.71	81.67	18.67	[6]
Eicosane	Biochars obtained by the pyrolysis of softwood and wheat straw at 550 °C and 700 °C	Softwood biochar/eicosane–37.0 Wheat straw biochar/eicosane—37.0	N/A	Softwood biochar/eicosane–53.4 Wheat straw biochar/eicosane–75.0	N/A	[47]
n-Eicosane	Natural rubber-latex	35–45	20–35	encap-C20 prepared at polymer weight contents of 20 wt.%: 204.5 ± 8.5 and encap-C20 prepared at polymer weight contents of 9 wt.% 239.8 ± 3.8	encap-C20 prepared at polymer weight contents of 20 wt.%:208.2 ± 9.4 and encap-C20 prepared at polymer weight contents of 9 wt.% 251.4 ± 6.2 J g_1	[48]
n-Octacosane	κ-Carrageenan	63.6	157.7	51.1	249.3	[49]
n-Octadecane	Aqueous silk solution	22.95 ± 60.73	4.63 ± 60.38	34.98 ± 61.40	37.58 ± 62.72	[50]
1-Octadecanol, 1-Eicosanol, 1-Docosanol	Acrylated soybean oil (ASO) F2—(ASO/1- Octadecanol) F3—(ASO/1- Eicosanol) F2—(ASO/1- Docosanol)	F2—60.46 F3—66.67 F2—70.07	F2—30.06 F3—45.58 F2—67.51	F2—49.39 F3—56.18 F2—60.82	F2—−17.56 F3—−41.01 F2—−70.07	[51]
Capric acid	Silkworm cocoons	30.6	123.4	26.7	122.6	[52]
Myristic acid	Orange peel biochar	55.37	67.20	49	65.14	[53]
Vegetable-derived PA	Bio-based PLA	62.3	N/A	59.9	N/A	[54]
CA and LA	SA	16.54	35.18	11.84	N/A	[55]
Sugarcane wax/Al_2_O_3_ composite	Gelatin, gum Arabic	68.00	61.00	59.66	44.45	[56]
Paraffin wax type Heptacosane	Waste chicken feathers	77.58	N/A	156.56	N/A	[57]
Beeswax	Coffee grounds collected after brewing in an automatic coffee machine	50.1	121.08	34.23	129.36	[58]
Sodium sulfate decahydrate-Na_2_SO_4_·10H_2_O	SA	32.6	N/A	37.1	N/A	[59]
Lignin-g-PCL copolymers	Alkaline lignin	51.33	N/A	61.16	N/A	[60]
Erythritol (ET)-grafted-PLA	Meso-erythritol, PLA	163.8	170.7	N/A	N/A	[61]
Microencapsulated PCM (mixtures of branched-chain hydrocarbons encapsulated in melamine formaldehyde shell)	Microcrystalline cellulose 20 μm	N/A	130.34	N/A	N/A	[62]
Microcapsule PCMs (paraffins encapsulated in melamine shells)	PCM microcapsules on wool fabric	N/A	enthalpy after 10 washing cycles—2.92	N/A	N/A	[63]
Microencapsulated PCMs (paraffin in PMMA shells)	Bacterial cellulose (BC)	23.75	6.73	22.58	6.58	[64]
Microencapsulated PCM (mixtures of branched-chain hy-drocarbons encapsu-lated in melamine formaldehyde shell)	Pullulan	29.36	104.85	16.02/18.82	103.58	[65]

**Table 3:** Temperatures and heat of melting of PEG and their mixtures. Reprinted from [73] with permission from Wiley.

Sample	Heating Cycle	Melting Point [°C]	ΔH [J/g]
PEG 1000	1 2	44.2 40.0	165.3 168.6
PEG 3400	1 2 (2 peaks)	63.0 56.4/61.9	191.7 171.6
PEG 10,000	1 2	66.6 66.2	188.7 180.6
PEG 20,000	1 2	68.5 67.7	177.4 165.0
PEG 35,000	1 2	71.1 68.3	199.7 183.4
PEG 3400/PEG 10,000	1 2 (2 peaks)	66.4 55.6 64.3	169.2 173.5
PEG 1000/PEG 10,000	1 2 (2 peaks)	36.5 60.8 35.3	161.3 156.8
PEG 1000/PEG 3400	1 (2 peaks) 2 (3 peaks)	33.5 60.0 34.3 51.3 59.1	199.7 183.4

**Table 4:** Temperature, melting, and freezing heat of the tested polyethers determined by the DSC method (heating and cooling rate 10 °C/min). Reprinted from [75] with permission from Wiley.

Polyether	Melting			Freezing		
	Melting Range [°C]	T_max_ [°C]	Heat of Fusion [J/g]	Freezing Range [°C]	T_max_ [°C]	Heat of Freezeing [J/g]
PEG 3400	51.9 ÷ 65.2	63.4	166.8	30.4 ÷ 39.0	36.7	158.3
PPO 4000	−11.5 ÷ −6.0	−9.2	0.6	-	-	-
PTHF 2900	23.9 ÷ 33.7	29.7	91.0	−3.8 ÷ 5.7	0.2	85.4

**Table 5:** The advantages and disadvantages of the encapsulation techniques. Reprinted from [76] with permission from Elsevier.

Advantages	Disadvantages
Protection from light, heat, moisture, and high oxygen concentration that might lead to the decomposition of PCMs	Micro and nanoencapsulation are complicated to conduct, and macro encapsulation has a lower structure stability and fracture resistance
Prevent evaporation of volatile compounds that harm the environment and users’ health	Needs a standard selection procedure of PCMs and shell materials
Cover unpleasant odours	
Control the speeds of internal materials’ release	Low thermal conductivity due to the polymer used as the shell

## Data Availability

Not applicable.

## Abbreviations

BC	bacterial cellulose nanofibers
CA	capric acid
CAC	cellulose acetate
CCY	cotton yarn
CEL	cellulose
CET	methylhydroxycellulose
CMC	carboxymethylcellulose
CNFs	chitin nanofibers
CNT	carbon nanotubes
CS	chitosan
DCM	dichloromethane
DSC	differential scanning calorimetry
EC	ethyl cellulose
GO	graphene oxide
HPMC	hydroxylpropyl methyl cellulose
LA	lauric acid
MA	myristic acid
MC	methyl cellulose
PA	palmitic acid
PAN	polyacrylonitrile
PBAT	poly(butylene adipate-co-butylene terephthalate)
PBSA	poly(butylene succinate-co-butylene adipate)
PCL	poly(ε-caprolactone)
PCM	phase-change material
PDA	polydopamine
PEF	poly(ethylene furanoate)
PEG	poly(ethylene glycol)
PEO	poly(ethylene oxide)
PET	poly(ethylene terephthalate)
PGI	poly(glycerol-itaconic acid)
PLA	poly(lactic acid)
POM	polyoxymethylene
PPO	poly(propylene oxide)
PTHF	polytetrahydrofurane
PTT	poly(trimethylene terephthalate)
PVA	poly(vinyl alcohol)
SF	silk fibroin
TD	tetradecanol

## References

[1] Pielichowska K., Pielichowski K. (2014). Phase Change Materials for Thermal Energy Storage. Prog. Mater. Sci..

[2] Zalba B., Marin J.M., Cabeza L.F., Mehling H. (2003). Review on Thermal Energy Storage with Phase Change: Materials, Heat Transfer Analysis and Applications. Appl. Therm. Eng..

[3] Pielichowska K., Pielichowski K. (2023). Multifunctional Phase Change Materials.

[4] Confalonieri C., Gariboldi E., Pielichowska K., Pielichowski K. (2023). 9—Shape-Stabilized and Form-Stable PCMs. Multifunctional Phase Change Materials.

[5] Pielichowska K., Bieda J., Szatkowski P. (2016). Polyurethane/Graphite Nano-Platelet Composites for Thermal Energy Storage. Renew. Energy.

[6] Moghaddam M.K., Mortazavi S.M., Khayamian T. (2015). Preparation of Calcium Alginate Microcapsules Containing N-Nonadecane by a Melt Coaxial Electrospray Method. J. Electrost..

[7] Pielichowska K., Pielichowski K. (2010). Novel Biodegradable Form Stable Phase Change Materials: Blends of Poly(Ethylene Oxide) and Gelatinized Potato Starch. J. Appl. Polym. Sci..

[8] Okogeri O., Stathopoulos V.N. (2021). What about Greener Phase Change Materials? A Review on Biobased Phase Change Materials for Thermal Energy Storage Applications. Int. J. Thermofluids.

[9] Masutani K., Kimura Y., Kobayashi S., Müllen K. (2021). Biobased Polymers BT—Encyclopedia of Polymeric Nanomaterials.

[10] Piorkowska E., Di Lorenzo M.L., Androsch R. (2019). Overview of Biobased Polymers. Thermal Properties of Bio-Based Polymers.

[11] Nakajima H., Dijkstra P., Loos K. (2017). The Recent Developments in Biobased Polymers toward General and Engineering Applications: Polymers That Are Upgraded from Biodegradable Polymers, Analogous to Petroleum-Derived Polymers, and Newly Developed. Polymers.

[12] Narayan R. (1988). Preparation of Bio-Based Polymers for Materials Applications. Appl. Biochem. Biotechnol..

[13] Carafa R.N., Foucher D.A., Sacripante G.G. (2023). Biobased Polymers from Lignocellulosic Sources. Green Chem. Lett. Rev..

[14] Kimura Y. (2009). Molecular, Structural, and Material Design of Bio-Based Polymers. Polym. J..

[15] Hatti-Kaul R., Nilsson L.J., Zhang B., Rehnberg N., Lundmark S. (2020). Designing Biobased Recyclable Polymers for Plastics. Trends Biotechnol..

[16] Mtibe A., Motloung M.P., Bandyopadhyay J., Ray S.S. (2021). Synthetic Biopolymers and Their Composites: Advantages and Limitations—An Overview. Macromol. Rapid Commun..

[17] https://plasticseurope.org/knowledge-hub/plastics-the-facts-2022/.

[18] https://www.cas.org/resources/cas-insights/materials/biopolymers-manufacturings-latest-green-hero.

[19] https://www.european-bioplastics.org/market/.

[20] https://bioplasticsnews.com/top-bioplastics-producers/.

[21] https://biokunststofftool.de/materials/bio-pp/?lang=en.

[22] https://natureplast.eu/en/matiere/biobased-pa-2/.

[23] Wijesena R.N., Tissera N.D., Rathnayaka V.W.S.G., Rajapakse H.D., de Silva R.M., de Silva K.M.N. (2020). Shape-Stabilization of Polyethylene Glycol Phase Change Materials with Chitin Nanofibers for Applications in “Smart” Windows. Carbohydr. Polym..

[24] Chen X., Zhao Y., Zhang Y., Lu A., Li X., Liu L., Qin G., Fang Z., Zhang J., Liu Y. (2019). A Novel Design and Synthesis of Multifunctional Magnetic Chitosan Microsphere Based on Phase Change Materials. Mater. Lett..

[25] Li J., Meng L., Chen J., Chen X., Wang Y., Xiao Z., Wang H., Liang D., Xie Y. (2023). Encapsulation of Polyethylene Glycol in Cellulose-Based Porous Capsules for Latent Heat Storage and Light-to-Thermal Conversion. Front. Chem. Sci. Eng..

[26] Niu L., Li X., Zhang Y., Yang H., Feng J., Liu Z. (2022). Electrospun Lignin-Based Phase-Change Nanofiber Films for Solar Energy Storage. ACS Sustain. Chem. Eng..

[27] Baniasadi H., Madani M., Seppälä J., Zimmerman J.B., Yazdani M.R. (2023). Form-Stable Phase Change Electrospun Nanofibers Mat with Thermal Regulation and Biomedical Multi-Functionalities. J. Energy Storage.

[28] Li Y.-Q., Huang X., Li Y., Xi Z., Hai G., Tao Z., Wang G. (2020). Shape-Stabilized Phase-Change Materials Supported by Eggplant-Derived Porous Carbon for Efficient Solar-to-Thermal Energy Conversion and Storage. Sustain. Energy Fuels.

[29] Feng L., Ding J., Hu H., Lv Z., Zhang Y., Xu B., Quan J., Hao S., Fan H., Hang Z. (2023). Preparation and Characterization of Bio-Based PLA/PEG/g-C3N4 Low-Temperature Composite Phase Change Energy Storage Materials. Polymers.

[30] Sheng X., Dong D., Lu X., Zhang L., Chen Y. (2020). MXene-Wrapped Bio-Based Pomelo Peel Foam/Polyethylene Glycol Composite Phase Change Material with Enhanced Light-to-Thermal Conversion Efficiency, Thermal Energy Storage Capability and Thermal Conductivity. Compos. Part A Appl. Sci. Manuf..

[31] Wei D., Wu C., Jiang G., Sheng X., Xie Y. (2021). Lignin-Assisted Construction of Well-Defined 3D Graphene Aerogel/PEG Form-Stable Phase Change Composites towards Efficient Solar Thermal Energy Storage. Sol. Energy Mater. Sol. Cells.

[32] Le W.T., Kankkunen A., Rojas O.J., Yazdani M.R. (2023). Leakage-Free Porous Cellulose-Based Phase Change Cryogels for Sound and Thermal Insulation. Sol. Energy Mater. Sol. Cells.

[33] Baniasadi H., Seppälä J., Kankkunen A., Seppälä A., Yazdani M.R. (2023). Water-Resistant Gum-Based Phase Change Composite for Thermo-Regulating Insulation Packaging. J. Energy Storage.

[34] Liu L., Fan X., Zhang Y., Zhang S., Wang W., Jin X., Tang B. (2020). Novel Bio-Based Phase Change Materials with High Enthalpy for Thermal Energy Storage. Appl. Energy.

[35] Yin G.Z., Díaz Palencia J.L., Wang D.Y. (2021). Fully Bio-Based Poly (Glycerol-Itaconic Acid) as Supporter for PEG Based Form Stable Phase Change Materials. Compos. Commun..

[36] Ke G., Jin X., Cai G., Li W., Xu A. (2022). A Novel Composite Cotton Yarn with Phase Change and Electrical Conductivity Functions. J. Ind. Text..

[37] Xie Y., Li W., Huang H., Dong D., Zhang X., Zhang L., Chen Y., Sheng X., Lu X. (2020). Bio-Based Radish@PDA/PEG Sandwich Composite with High Efficiency Solar Thermal Energy Storage. ACS Sustain. Chem. Eng..

[38] Chen C., Zhao Y., Liu W. (2013). Electrospun Polyethylene Glycol/Cellulose Acetate Phase Change Fibers with Core-Sheath Structure for Thermal Energy Storage. Renew. Energy.

[39] Zhang H.-C., Kang B.-h., Sheng X., Lu X. (2019). Novel Bio-Based Pomelo Peel Flour/Polyethylene Glycol Composite Phase Change Material for Thermal Energy Storage. Polymers.

[40] Pielichowska K., Pielichowski K. (2011). Biodegradable PEO/Cellulose-Based Solid-Solid Phase Change Materials. Polym. Adv. Technol..

[41] Liu Y., Wu Y., Zhao S., Wang X., Zheng J., Zeng W., Yuan M., Zhao N., Li Q., Wang Z. (2023). Biobased Phase Change Material with Reduced Thermal Conductivity: From Preparation to Analysis of Thermal Insulation Performance. ACS Appl. Polym. Mater..

[42] Singh J., Vennapusa J.R., Dixit P., Maiti T.K., Chattopadhyay S. (2022). A Novel Strategy for Temperature Controlling of Chocolates through 1-Dodecanol Embedded Polyurea Coated Barium Alginate Beads. J. Taiwan Inst. Chem. Eng..

[43] Atinafu D.G., Yang S., Yun B.Y., Kang Y., Kim S. (2022). Use of Biochar Co-Mediated Chitosan Mesopores to Encapsulate Alkane and Improve Thermal Properties. Environ. Res..

[44] Montanari C., Chen H., Lidfeldt M., Gunnarsson J., Olsén P., Berglund L.A. (2023). Sustainable Thermal Energy Batteries from Fully Bio-Based Transparent Wood. Small.

[45] Qu M., Guo C., Li L., Zhang X. (2017). Preparation and Investigation on Tetradecanol and Myristic Acid/Cellulose Form-Stable Phase Change Material. J. Therm. Anal. Calorim..

[46] Reddy V.J., Dixit P., Singh J., Chattopadhyay S. (2022). Understanding the Core-Shell Interactions in Macrocapsules of Organic Phase Change Materials and Polysaccharide Shell. Carbohydr. Polym..

[47] Atinafu D.G., Yeol Yun B., Uk Kim Y., Wi S., Kim S. (2021). Introduction of Eicosane into Biochar Derived from Softwood and Wheat Straw: Influence of Porous Structure and Surface Chemistry. Chem. Eng. J..

[48] Phadungphatthanakoon S., Poompradub S., Wanichwecharungruang S.P. (2011). Increasing the Thermal Storage Capacity of a Phase Change Material by Encapsulation: Preparation and Application in Natural Rubber. ACS Appl. Mater. Interfaces.

[49] Jin L., Tan Y., Yuan S., Wang S., Cheng X., Wang H., Du Z., Du X. (2023). Phytic Acid–Decorated κ-Carrageenan/Melanin Hybrid Aerogels Supported Phase Change Composites with Excellent Photothermal Conversion Efficiency and Flame Retardancy. Renew. Energy.

[50] Zhao L., Luo J., Li Y., Wang H., Song G., Tang G. (2017). Emulsion-Electrospinning n-Octadecane/Silk Composite Fiber as Environmental-Friendly Form-Stable Phase Change Materials. J. Appl. Polym. Sci..

[51] Baştürk E., Kahraman M.V. (2016). Photocrosslinked Biobased Phase Change Material for Thermal Energy Storage. J. Appl. Polym. Sci..

[52] Wang C., Dong H., Cheng C., Sun K., Jin T., Shi Q. (2022). Flexible and Biocompatible Silk Fiber-Based Composite Phase Change Material for Personal Thermal Management. ACS Sustain. Chem. Eng..

[53] Mandal S., Ishak S., Lee D.-E., Park T. (2022). Shape-Stabilized Orange Peel/Myristic Acid Phase Change Materials for Efficient Thermal Energy Storage Application. Energy Rep..

[54] Fashandi M., Leung S.N. (2017). Preparation and Characterization of 100% Bio-Based Polylactic Acid/Palmitic Acid Microcapsules for Thermal Energy Storage. Mater. Renew. Sustain. Energy.

[55] M’ghari O., Hassani F.S.A., Mekhzoum M.E.M., Zari N., Bouhfid R., el Kacem Qaiss A. (2021). Elaboration of a Composite Material Based on Plaster Reinforced with Phase Change Material/Oakum Fiber: Physical, Thermal and Mechanical Properties. J. Energy Storage.

[56] Tangsiriratana E., Skolpap W., Patterson R.J., Sriprapha K. (2019). Thermal Properties and Behavior of Microencapsulated Sugarcane Wax Phase Change Material. Heliyon.

[57] Abdulmunem A.R., Samin P.M., Sopian K., Hoseinzadeh S., Al-Jaber H.A., Garcia D.A. (2022). Waste Chicken Feathers Integrated with Phase Change Materials as New Inner Insulation Envelope for Buildings. J. Energy Storage.

[58] Souissi M., Trigui A., Jedidi I., Loukil M.S., Abdelmouleh M. (2023). Bio-Based Composite as Phase Change Material Including Spent Coffee Grounds and Beeswax Paraffin. Korean J. Chem. Eng..

[59] Meng L., Ivanov A.S., Kim S., Zhao X., Kumar N., Young-Gonzales A., Saito T., Bras W., Gluesenkamp K., Bocharova V. (2022). Alginate-Sodium Sulfate Decahydrate Phase Change Composite with Extended Stability. ACS Appl. Polym. Mater..

[60] Lee J.J.C., Sugiarto S., Ong P.J., Soo X.Y.D., Ni X., Luo P., Hnin Y.Y.K., See J.S.Y., Wei F., Zheng R. (2022). Lignin-g-Polycaprolactone as a Form-Stable Phase Change Material for Thermal Energy Storage Application. J. Energy Storage.

[61] Lee W., Lee J., Yang W., Kim J. (2023). Fabrication of Biobased Advanced Phase Change Material and Multifunctional Composites for Efficient Thermal Management. ACS Sustain. Chem. Eng..

[62] Ahn Y.-H., DeWitt S.J.A., McGuire S., Lively R.P. (2021). Incorporation of Phase Change Materials into Fibers for Sustainable Thermal Energy Storage. Ind. Eng. Chem. Res..

[63] Oliveira F.R., Fernandes M., Carneiro N., Pedro Souto A. (2013). Functionalization of Wool Fabric with Phase-Change Materials Microcapsules after Plasma Surface Modification. J. Appl. Polym. Sci..

[64] Pinto S.C., Silva N.H.C.S., Pinto R.J.B., Freire C.S.R., Duarte I., Vicente R., Vesenjak M., Marques P.A.A.P. (2020). Multifunctional Hybrid Structures Made of Open-Cell Aluminum Foam Impregnated with Cellulose/Graphene Nanocomposites. Carbohydr. Polym..

[65] Kizildag N. (2023). Pullulan Films with PCMs: Recyclable Bio-Based Films with Thermal Management Functionality. Coatings.

[66] Kamimoto M., Abe Y., Sawata S., Tani T., Ozawa T. (1986). Latent Thermal Storage Unit Using Form-Stable High Density Polyethylene; Part I: Performance of the Storage Unit. J. Sol. Energy Eng..

[67] Weingrill H.M., Resch-Fauster K., Lucyshyn T., Zauner C. (2019). High-Density Polyethylene as Phase-Change Material: Long-Term Stability and Aging. Polym. Test..

[68] Weingrill H.M., Resch-Fauster K., Zauner C. (2018). Applicability of Polymeric Materials as Phase Change Materials. Macromol. Mater. Eng..

[69] Iwamoto Y., Ikai S. New Polymeric Material for Latent Heat Thermal Energy Storage. Proceedings of the 5th Workshop of the IEA ECES IA Annex 10.

[70] Herzberger J., Niederer K., Pohlit H., Seiwert J., Worm M., Wurm F.R., Frey H. (2016). Polymerization of Ethylene Oxide, Propylene Oxide, and Other Alkylene Oxides: Synthesis, Novel Polymer Architectures, and Bioconjugation. Chem. Rev..

[71] Bailey F.E., Koleske J.V. (1976). Poly(Ethylene Oxide).

[72] Pielichowski K., Flejtuch K. (2003). Differential Scanning Calorimetry Study of Blends of Poly(Ethylene Glycol) with Selected Fatty Acids. Macromol. Mater. Eng..

[73] Pielichowski K., Flejtuch K. (2002). Differential Scanning Calorimetry Studies on Poly(Ethylene Glycol) with Different Molecular Weights for Thermal Energy Storage Materials. Polym. Adv. Technol..

[74] Flejtuch K. (2004). Badanie Przemian Fazowych Wybranych Ukladów Polieterów Pod Kątem Akumulacji Energii Cieplnej. PhD Thesis.

[75] Pielichowski K., Flejtuch K. (2003). Binary Blends of Polyethers with Fatty Acids: A Thermal Characterization of the Phase Transitions. J. Appl. Polym. Sci..

[76] Huang Y., Stonehouse A., Abeykoon C. (2023). Encapsulation Methods for Phase Change Materials—A Critical Review. Int. J. Heat Mass Transf..

[77] Ghasemi K., Tasnim S., Mahmud S. (2022). PCM, Nano/Microencapsulation and Slurries: A Review of Fundamentals, Categories, Fabrication, Numerical Models and Applications. Sustain. Energy Technol. Assess..

[78] Deveci S.S., Basal G. (2009). Preparation of PCM Microcapsules by Complex Coacervation of Silk Fibroin and Chitosan. Colloid Polym. Sci..

[79] Jeon I.K., Azzam A., Al Jebaei H., Kim Y.-R., Aryal A., Baltazar J.-C. (2023). Effects of Shape-Stabilized Phase Change Materials in Cementitious Composites on Thermal-Mechanical Properties and Economic Benefits. Appl. Therm. Eng..

